# Aortic Ring Assay

**DOI:** 10.3791/1564

**Published:** 2009-11-24

**Authors:** Keren Bellacen, Eli C. Lewis

**Affiliations:** Department Clinical Biochemistry, Ben-Gurion University

## Abstract

Angiogenesis, the sprouting of blood vessels from preexisting vasculature is associated with both natural and pathological processes. Various angiogenesis assays involve the study of individual endothelial cells in culture conditions (1). The aortic ring assay is an angiogenesis model that is based on organ culture. In this assay, angiogenic vessels grow from a segment of the aorta (modified from (2)). Briefly, mouse thoracic aorta is excised, the fat layer and adventitia are removed, and rings approximately 1 mm in length are prepared. Individual rings are then embedded in a small solid dome of basement matrix extract (BME), cast inside individual wells of a 48-well plate. Angiogenic factors and inhibitors of angiogenesis can be directly added to the rings, and a mixed co-culture of aortic rings and other cell types can be employed for the study of paracrine angiogenic effects. Sprouting is observed by inspection under a stereomicroscope over a period of 6-12 days. Due to the large variation caused by the irregularities in the aortic segments, experimentation in 6-plicates is strongly advised. Neovessel outgrowth is monitored throughout the experiment and imaged using phase microscopy, and supernatants are collected for measurement of relevant angiogenic and anti-angiogenic factors, cell death markers and nitrite.

**Figure:**
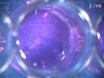


## Protocol

### One day before the experiment:

1. Allow BME (reduced growth factor, Cultrex, Trevigen) to thaw on ice or at 4°C; once thawed keep on ice to prevent re-solidifying.

### Day of the experiment:

1. Mice (6-7 weeks old) are to be anesthetized and bled out.
2. Remove thoracic aorta into a Petri dish filled with cold sterile PBS.
3. Perform mechanical cleaning from surrounding fat tissue. Note, avoid drying of Aorta at any time.
4. Using a surgical blade, slice Aorta evenly into 1 mm rings. The cut must be uniform and clean.
5. Transfer rings to fresh cold PBS and keep on ice at all times.
6. Using pre-cooled pipette tips, add to each well in a 48-well plate a rounded drop of 150 ml cold BME. Allow the BME drop to solidify at 37°C for 20-30 minutes.
7. Place a single aortic ring in the top center of each dome. Incubate for 10 minutes at 37°C.
8. On top of each ring add an additional 150 ml BME. Incubate for 20-30 minutes at 37°C.
9. Prepare medium: Human endothelial serum free medium (GIBCO, Carlsbad, CA) supplemented with 2% FCS, 50 units/ml penicillin and 50 μg/ml streptomycin (Cellgro).
10. To each well add 500 ml medium containing either:
  1. Experimental samples.
  2. Positive control: Endothelial cell growth supplement (ECGS, final concentration 200 μg/ml, BD Biosciences).
  3. Negative control: Medium alone.
11. Incubate plate at 37°C for 12 days, photograph rings between days 6 and 12 under standard phase contrast stereoscope.
12. Optional: Collect supernatants.

### Notes:

1. Keep sterile conditions at all times.
2. Avoid introduction of bubbles into the BME.

## Discussion

Aortic ring assay is a beneficial tool on the way to evaluate angiogenic as well as anti-angiogenic factors. The vessels that grow out from aortic rings recruit smooth muscle cells and pericytes to associate with the endothelial cell tube, meaning that they are anatomically similar to neovessels *in vivo*. Furthermore, the mouse aortic ring assay holds the advantage of the vast array of transgenic tools available for this species. Yet, there are several disadvantages to the aortic ring assay. First, vessels outgrowth *in vivo* occurs from microvessels and not from major vessels such as the aorta. Second, inconsistency in the handling of the rings and the amount of surrounding tissue remaining on the vessel can influence vessel outgrowth. Rings from different aortas or different mice's ages and strains can also interfere with the angiogenic responses. And lastly, Angiogenic vessel outgrowth occurs in three dimensions, which makes it non-ideal to photograph and quantify. Taken together, when performed with sufficient internal controls and with multiple repeats, this assay is relatively simple, non-expensive, highly informative and significantly superior to endothelial cultures in its biological complexity and relevance.

## References

[B0] Goodwin AM (2007). In vitro assays of angiogenesis for assessment of angiogenic and anti-angiogenic agents. Microvascular Research.

[B1] Masson V, Devy L, Grignet-Debrus C, Bernt S, Bajou K, Blacher S, Roland G, Chang Y, Fong T, Carmeliet P, Foidart JM, Noel A (2002). Mouse aortic ring assay: a new approach of the molecular genetics of angiogenesis. Biol. Proced. Online.

